# Hybrid proximal linearized algorithm for the split DC program in infinite-dimensional real Hilbert spaces

**DOI:** 10.1186/s13660-018-1840-6

**Published:** 2018-09-21

**Authors:** Chih-Sheng Chuang, Pei-Jung Yang

**Affiliations:** Department of Applied Mathematics, National Chiayi University, Chiayi, Taiwan

**Keywords:** 49J50, 49J53, 49M30, 49M37, DC function, Subdifferential, Strongly monotonicity, Critical point

## Abstract

To be the best of our knowledge, the convergence theorem for the DC program and split DC program are proposed in finite-dimensional real Hilbert spaces or Euclidean spaces. In this paper, to study the split DC program, we give a hybrid proximal linearized algorithm and propose related convergence theorems in the settings of finite- and infinite-dimensional real Hilbert spaces, respectively.

## Introduction

Let *H* be a real Hilbert space, and let $f:H\rightarrow \mathbb{R}$ be a proper lower semicontinuous and convex function. Define a sequence $\{x_{n}\}_{n\in\mathbb{N}}$ by taking $x_{1}\in H$ arbitrarily and

1.1 $$ x_{n+1}=\operatorname{arg}\min_{y\in H} \biggl\{ f(y)+\frac{1}{2\beta _{n}} \Vert y-x_{n} \Vert ^{2} \biggr\} ,\quad n\in\mathbb{N}. $$

Then $\{x_{n}\}_{n\in\mathbb{N}}$ converges weakly to a minimizer of *f* under suitable conditions, and this is called the proximal point algorithm (PPA). This algorithm is useful, however, only for convex problems, because the idea for this algorithm is based on the monotonicity of subdifferential operators of convex functions. So, it is important to consider the relation between nonconvex functions and proximal point algorithm.

The DC program is the well-known nonconvex problem of the form

$$(\mathrm{DCP})\quad \text{Find }\bar{x}\in\arg\min_{x\in\mathbb{R}^{n}}\bigl\{ f(x)=g(x)-h(x)\bigr\} , $$

where $g,h:\mathbb{R}^{n}\rightarrow \mathbb{R}$ are proper lower semicontinuous convex functions. Here, the function *f* is called a DC function, and the functions *g* and *h* are called the DC components of *f*. (In the DC program, the convention $(+\infty)-(+\infty)=+\infty $ is adopted to avoid the ambiguity $(+\infty)-(+\infty)$ that does not present any interest.) It is well known that a necessary condition for $x\in\operatorname{dom}(f):=\{x\in\mathbb{R}^{n}: (x)<\infty\}$ to be a local minimizer of *f* is $\partial h(x)\subseteq \partial g(x)$. However, this condition is hard to be reached. So, many researchers focus their attentions on finding points such that $\partial h(x)\cap \partial g(x)\neq\emptyset$, where *x* is called a critical point of *f* [1].

It is worth mentioning the richness of the class of DC functions that is a subspace containing the class of lower-$\mathcal{C}^{2}$ functions. In particular, $\mathcal{DC}(\mathbb{R}^{n})$ contains the space $\mathcal{C}^{1,1}$ of functions with locally Lipschitz continuous gradients. Further, $\mathcal{DC}(\mathbb{R}^{n})$ is closed under the operations usually considered in optimization. For example, a linear combination, a finite supremum, or the product of two DC functions remain DC. It is also known that the set of DC functions defined on a compact convex set of $\mathbb{R}^{n}$ is dense in the set of continuous functions on this set.

The interest in the theory of DC functions has much increased in the last years. Some interesting optimality conditions and duality theorems related to the DC program are given. For more details, we refer to [2–9].

In 2003, Sun, Sampaio, and Candido [10] proposed a proximal point algorithm to study problem (DCP).

### Algorithm 1.1

(Proximal point algorithm for (DCP) [10])

Let $\{\beta_{n}\}_{n\in \mathbb{N}}$ be a sequence in $(0,\infty)$, and let $g,h:\mathbb {R}^{k}\rightarrow \mathbb{R}$ be proper lower semicontinuous and convex functions. Let $\{x_{n}\}_{n\in\mathbb{N}}$ be generated as follows:

$$\textstyle\begin{cases} x_{1}\in H_{1}\text{ is chosen arbitrarily},\\ \text{Compute }w_{n}\in\partial h(x_{n})\text{ and set }y_{n}=x_{n}+\beta _{n}w_{n},\\ x_{n+1}:=(I+\beta_{n}\partial g)^{-1}(y_{n}),\quad n\in\mathbb{N}.\\ \text{Stop criteria: } x_{n+1}=x_{n}. \end{cases} $$

In 2016, Souza, Oliveira, and Soubeyran [11] proposed a proximal linearized algorithm to study the DC program.

### Algorithm 1.2

(Proximal linearized algorithm [11])

Let $\{\beta_{n}\}_{n\in \mathbb{N}}$ be a sequence in $(0,\infty)$, and let $g,h:\mathbb {R}^{k}\rightarrow \mathbb{R}$ be proper lower semicontinuous and convex functions. Let $\{x_{n}\}_{n\in\mathbb{N}}$ be generated as follows:

$$\textstyle\begin{cases} x_{1}\in H_{1}\text{ is chosen arbitrarily},\\ \text{Compute }w_{n}\in\partial h(x_{n}),\\ x_{n+1}:=\arg\min_{u\in H_{1}}\{g(u)+\frac{1}{2\beta _{n}} \Vert u-x_{n} \Vert ^{2}-\langle w_{n},u-x_{n}\rangle\}, \quad n\in\mathbb{N}.\\ \text{Stop criteria: } x_{n+1}=x_{n}. \end{cases} $$

Besides, some algorithms for the DC program are proposed to analyze and solve a variety of highly structured and practical problems (see, for example, [12]).

On the other hand, Chuang [13] introduced the following split DC program (split minimization problems for DC functions):

$$ (\text{SDCP})\quad \text{Find } \bar{x}\in H_{1} \text{ such that }\bar{x}\in\arg\min_{x\in H_{1}}f_{1}(x)\text{ and } A\bar{x}\in\arg\min_{y\in H_{2}}f_{2}(y), $$

where $H_{1}$ and $H_{2}$ are real Hilbert spaces, $A:H_{1}\rightarrow H_{2}$ is a linear bounded mapping with adjoint $A^{*}$, $g_{1},h_{1}:H_{1}\rightarrow \mathbb{R}$ and $g_{2},h_{2}:H_{2}\rightarrow \mathbb{R}$ are proper lower semicontinuous and convex functions, and $f_{1}(x)=g_{1}(x)-h_{1}(x)$ and $f_{2}(y)=g_{2}(y)-h_{2}(y)$ for all $x\in H_{1}$ and $y\in H_{2}$. Further, to study problem (SDCP), Chuang [13] gave the following split proximal linearized algorithm and related convergence theorem in finite-dimensional real Hilbert spaces.

### Algorithm 1.3

(Split proximal linearized algorithm)

Let $\{x_{n}\}_{n\in\mathbb{N}}$ be generated as follows:

$$\textstyle\begin{cases} x_{1}\in H_{1}\text{ is chosen arbitrarily},\\ y_{n}:=\arg\min_{v\in H_{2}}\{g_{2}(v)+\frac{1}{2\beta _{n}} \Vert v-Ax_{n} \Vert ^{2}-\langle\nabla h_{2}(Ax_{n}),v-Ax_{n}\rangle\},\\ z_{n}:=x_{n}-r_{n} A^{*}(Ax_{n}-y_{n}),\\ x_{n+1}:=\arg\min_{u\in H_{1}}\{g_{1}(u)+\frac{1}{2\beta _{n}} \Vert u-z_{n} \Vert ^{2}-\langle\nabla h_{1}(z_{n}),u-z_{n}\rangle\},\quad n\in\mathbb{N}. \end{cases} $$

Besides, there are also some important algorithms for the related problems in the literature; see, for example, [14–17].

In this paper, motivated by the works mentioned, we first give an hybrid proximal linearized algorithm and then propose a related convergence theorem in finite-dimensional real Hilbert spaces. Next, we propose related convergence theorems in infinite-dimensional real Hilbert space.

## Preliminaries

Let *H* be a real Hilbert space with inner product $\langle\cdot ,\cdot\rangle$ and norm $\|\cdot\|$. We denote the strong and weak convergence of $\{x_{n}\}_{n\in\mathbb{N}}$ to $x\in H$ by $x_{n}\rightarrow x$ and $x_{n}\rightharpoonup x$, respectively. For all $x,y,u,v\in H$ and $\lambda\in\mathbb{R}$, we have

2.1 $$\begin{aligned} & \Vert x+y \Vert ^{2}= \Vert x \Vert ^{2}+2\langle x,y\rangle+ \Vert y \Vert ^{2}, \end{aligned}$$

2.2 $$\begin{aligned} & \bigl\Vert \lambda x+(1-\lambda)y \bigr\Vert ^{2}= \lambda \Vert x \Vert ^{2}+(1-\lambda ) \Vert y \Vert ^{2}-\lambda(1-\lambda) \Vert x-y \Vert ^{2}, \end{aligned}$$

2.3 $$\begin{aligned} &2\langle x-y,u-v\rangle= \Vert x-v \Vert ^{2}+ \Vert y-u \Vert ^{2}- \Vert x-u \Vert ^{2}- \Vert y-v \Vert ^{2}. \end{aligned}$$

### Definition 2.1

Let *H* be a real Hilbert space, let $B:H\rightarrow H$, and let $\beta>0$. Then,

(i) *B* is monotone if $\langle x-y,Bx-By\rangle\geq0$ for all $x,y\in H$.
(ii) *B* is *β*-strongly monotone if $\langle x-y,Bx-By\rangle\geq\beta\|x-y\|^{2}$ for all $x,y\in H$.

### Definition 2.2

Let *H* be a real Hilbert space, and let $B:H\multimap H$ be a set-valued mapping with domain $\mathcal{D}(B):=\{x\in H:B(x)\neq \emptyset\}$. Then,

(i) *B* is monotone if $\langle u-v,x-y\rangle\geq0$ for any $u\in B(x)$ and $v\in B(y)$.
(ii) *B* is maximal monotone if its graph $\{(x,y):x\in \mathcal{D}(B), y\in B(x)\}$ is not properly contained in the graph of any other monotone mapping.
(iii) *B* is *ρ*-strongly monotone ($\rho>0$) if $\langle x-y,u-v\rangle\geq\rho\|x-y\|^{2}$ for all $x,y\in H$, $u\in B(x)$, and $v\in B(y)$.

### Definition 2.3

Let *H* be a real Hilbert space, and let $f:H\rightarrow\mathbb{R}$. Then,

(i) *f* is proper if $\operatorname{dom}(f)=\{x\in H:~f(x)<\infty\} \neq\emptyset$.
(ii) *f* is lower semicontinuous if $\{x\in H:f(x)\leq r\}$ is closed for each $r\in\mathbb{R}$.
(iii) *f* is convex if $f(tx+(1-t)y)\leq t f(x)+(1-t)f(y)$ for every $x,y\in H$ and $t\in[0,1]$.
(iv) *f* is *ρ*-strongly convex ($\rho>0$) if
  $$f\bigl(tx+(1-t)y\bigr)+\frac{\rho}{2}\cdot t(1-t) \Vert x-y \Vert ^{2}\leq tf(x)+(1-t)f(y) $$
  for all $x,y\in H$ and $t\in(0,1)$.
(v) *f* is Gâteaux differentiable at $x\in H$ if there is $\nabla f(x)\in H$ such that
  $$\lim_{t\rightarrow0}\frac{f(x+ty)-f(x)}{t}=\bigl\langle y, \nabla f(x)\bigr\rangle $$
  for each $y\in H$.
(vi) *f* is Fréchet differentiable at *x* if there is $\nabla f(x)$ such that
  $$\lim_{y\rightarrow0}\frac{f(x+y)-f(x)-\langle\nabla f(x),y\rangle }{ \Vert y \Vert }=0. $$

### Example 2.1

Let *H* be a real Hilbert space. Then $g(x):=\|x\|^{2}$ is a 2-strongly convex function.

### Example 2.2

Let $g(x):=\frac{1}{2}\langle Qx,x\rangle-\langle x,b\rangle$, where $Q\in\mathbb{R}^{n\times n}$ is a real symmetric positive definite matrix, and $b\in\mathbb{R}^{n}$. Then *g* is a strongly convex function.

### Definition 2.4

Let $f:H\rightarrow(-\infty,\infty]$ be a proper lower semicontinuous and convex function. Then the subdifferential *∂f* of *f* is defined by

$$\partial f(x):=\bigl\{ x^{*}\in H: f(x)+\bigl\langle y-x,x^{*}\bigr\rangle \leq f(y) \text{ for each }y\in H\bigr\} $$

for each $x\in H$.

### Lemma 2.1

([18, 19])

*Let* $f:H\rightarrow(-\infty,\infty]$ *be a proper lower semicontinuous and convex function*. *Then*:

(i) *∂f* *is a set*-*valued maximal monotone mapping*;
(ii) *f* *is Gâteaux differentiable at* $x\in\operatorname{int}(\operatorname{dom}(f))$ *if and only if* $\partial f(x)$ *consists of a single element*, *that is*, $\partial f(x)=\{\nabla f(x)\}$ [18, *Prop*. 1.1.10];
(iii) *A Fréchet differentiable function* *f* *is convex if and only if* ∇*f* *is a monotone mapping*.

### Lemma 2.2

([19, Example 22.3(iv)])

*Let* $\rho>0$, *let* *H* *be a real Hilbert space*, *and let* $f:H\rightarrow \mathbb{R}$ *be a proper lower semicontinuous and convex function*. *If* *f* *is* *ρ*-*strongly convex*, *then* *∂f* *is* *ρ*-*strongly monotone*.

### Lemma 2.3

([19, Prop. 16.26])

*Let* *H* *be a real Hilbert space*, *and let* $f:H\rightarrow (\infty ,\infty]$ *be a proper lower semicontinuous and convex function*. *Let* $\{ u_{n}\}_{n\in\mathbb{N}}$ *and* $\{x_{n}\}_{n\in\mathbb{N}}$ *be sequences in* *H* *such that* $u_{n}\in\partial f(x_{n})$ *for all* $n\in\mathbb{N}$. *Then if* $x_{n}\rightharpoonup x$ *and* $u_{n}\rightarrow u$, *then* $u\in \partial f(x)$.

### Lemma 2.4

([20])

*Let* *H* *be a real Hilbert space*, *let* $B:H\multimap H$ *be a set*-*valued maximal monotone mapping*, *and let* $\beta>0$. *The mapping* $J_{\beta}^{B}$ *defined by* $J_{\beta}^{B}(x):=(I+\beta B)^{-1}(x)$ *for* $x\in H$ *is a single*-*valued mapping*.

## Main results in finite-dimensional real Hilbert space

Let *ρ* and *L* be real numbers with $\rho>L>0$. Let $H_{1}$ and $H_{2}$ be finite-dimensional real Hilbert spaces, and let $A:H_{1}\rightarrow H_{2}$ be a nonzero linear and bounded mapping with adjoint $A^{*}$. Let $g_{1},h_{1}:H_{1}\rightarrow \mathbb{R}$ be proper lower semicontinuous and convex functions, let $g_{2},h_{2}:H_{2}\rightarrow \mathbb{R}$ be proper lower semicontinuous and convex functions, and let $f_{1}(x)=g_{1}(x)-h_{1}(x)$ for $x\in H_{1}$ and $f_{2}(y)=g_{2}(y)-h_{2}(y)$ for $y\in H_{2}$. Further, we assume that $f_{1}$ and $f_{2}$ are bounded from below, $h_{1}$ and $h_{2}$ are Fréchet differentiable, $\nabla h_{1}$ and $\nabla h_{2}$ are *L*-Lipschitz continuous, and $g_{1}$ and $g_{2}$ are *ρ*-strongly convex.

Choose $\delta\in(0,0.5)$, let *β* be a real number, and let $\{ \beta_{n}\}_{n\in\mathbb{N}}$ be a sequence in $\mathbb{R}$ such that

$$0< \beta,\quad \beta_{n}< \frac{1}{2\rho-L}. $$

Since $\rho>L>0$ and $\beta_{n}>0$, we have $\beta_{n}L<\beta_{n}\rho$, and then

$$0< \frac{1+\beta_{n}L}{1+2\beta_{n}\rho-\beta_{n}L}< 1. $$

Besides, we know that

$$1< 1+2\beta_{n}\rho-\beta_{n}L< 2, $$

which implies that

$$\frac{1}{2}< \frac{1}{1+2\beta_{n}\rho-\beta_{n}L}< \frac{1+\beta _{n}L}{1+2\beta_{n}\rho-\beta_{n}L}< 1. $$

Let $\{r_{n}\}_{n\in\mathbb{N}}$ be a sequence in $\mathbb{R}$, and let *r* be a real number with

$$\liminf_{n\rightarrow \infty}r_{n}>0 $$

and

$$0< r_{n},\quad r< \min \biggl\{ \frac{\sqrt[4]{1-2\delta}\cdot\sqrt{\beta _{n}(\rho-L)}}{\sqrt{2+2\beta_{n} L}\cdot \Vert A \Vert ^{2}},\frac{\sqrt{\delta }}{(2+\beta_{n}L) \Vert A \Vert ^{2}} \biggr\} . $$

Thus we have

$$r_{n}< \frac{\sqrt{\delta}}{(2+\beta_{n}L) \Vert A \Vert ^{2}}< \frac{3}{2 \Vert A \Vert ^{2}} $$

and

$$0< \frac{4(1+\beta_{n} L)\cdot \Vert A \Vert ^{4}\cdot r_{n}^{2}}{\sqrt{1-2\delta }}< 2\beta_{n}\rho-2\beta_{n}L. $$

So, we have

$$0< 1+\beta_{n}L+\frac{4(1+\beta_{n} L)\cdot \Vert A \Vert ^{4}\cdot r_{n}^{2}}{\sqrt {1-2\delta}}< 1+2\beta_{n}\rho- \beta_{n} L, $$

and then

$$0< \frac{1+\beta_{n}L}{1+2\beta_{n}\rho-\beta_{n} L}\cdot \biggl(1+\frac {4\cdot \Vert A \Vert ^{4}\cdot r_{n}^{2}}{\sqrt{1-2\delta}} \biggr)< 1. $$

Let $\Omega_{\mathrm{SDCP}}$ be defined by

$$\Omega_{\mathrm{SDCP}}:=\bigl\{ x\in H_{1}:\nabla h_{1}(x) \in\partial g_{1}(x), \nabla h_{2}(Ax)\in\partial g_{2}(Ax)\bigr\} . $$

We further assume that $\Omega_{\mathrm{SDCP}}\neq\emptyset$. The following result of Chuang [13] plays an important role in this paper.

### Lemma 3.1

([13])

*Under the assumptions in this section*, *let*

3.1 $$ \textstyle\begin{cases} y:=\arg\min_{v\in H_{2}}\{g_{2}(v)+\frac{1}{2\beta} \Vert v-Ax \Vert ^{2}-\langle \nabla h_{2}(Ax),v-Ax\rangle\},\\ z:=x-rA^{*}(Ax-y),\\ w:=\arg\min_{u\in H_{1}}\{g_{1}(u)+\frac{1}{2\beta} \Vert u-z \Vert ^{2}-\langle \nabla h_{1}(z),u-z\rangle\}. \end{cases} $$

*Then* $x\in\Omega_{\mathrm{SDCP}}$ *if and only if* $x=w$.

### Proposition 3.1

([13])

*If* $\rho>L$ *and* $\Omega_{\mathrm{SDCP}}\neq\emptyset$, *then the set* $\Omega_{\mathrm{SDCP}}$ *is a singleton*.

In this section, we propose the following algorithm to study the split DC program.

### Algorithm 3.1

Let $x_{1}\in H_{1}$ be arbitrary, and let $\{x_{n}\}_{n\in\mathbb {N}}$ be defined as follows:

$$\textstyle\begin{cases} y_{n}:=\arg\min_{v\in H_{2}}\{g_{2}(v)+\frac{1}{2\beta _{n}} \Vert v-Ax_{n} \Vert ^{2}-\langle\nabla h_{2}(Ax_{n}),v-Ax_{n}\rangle\},\\ z_{n}:=x_{n}-r_{n} A^{*}(Ax_{n}-y_{n}),\\ w_{n}:=\arg\min_{u\in H_{1}}\{g_{1}(u)+\frac{1}{2\beta _{n}} \Vert u-z_{n} \Vert ^{2}-\langle\nabla h_{1}(z_{n}),u-z_{n}\rangle\},\\ \widehat{y}_{n}:=\arg\min_{v\in H_{2}}\{g_{2}(v)+\frac{1}{2\beta _{n}} \Vert v-Aw_{n} \Vert ^{2}-\langle\nabla h_{2}(Aw_{n}),v-Aw_{n}\rangle\},\\ \widehat{z}_{n}:=w_{n}-r_{n} A^{*}(Aw_{n}-\widehat{y}_{n}),\\ D_{n}:=z_{n}-\widehat{z}_{n},\\ \alpha_{n}:=\frac{\langle x_{n}-w_{n},D_{n}\rangle}{ \Vert D_{n} \Vert ^{2}},\\ \widehat{x}_{n}:=x_{n}-\alpha_{n}D_{n},\\ x_{n+1}:=\arg\min_{u\in H_{1}}\{g_{1}(u)+\frac{1}{2\beta_{n}} \Vert u-\widehat {x}_{n} \Vert ^{2}-\langle\nabla h_{1}(\widehat{x}_{n}),u-\widehat{x}_{n}\rangle\} ,\quad n\in\mathbb{N},\\ \text{stop criteria: } x_{n}=w_{n}. \end{cases} $$

### Remark 3.1

The stop criteria in Algorithm 3.1 is given by Lemma 3.1.

### Theorem 3.1

*Let* $\{x_{n}\}_{n\in\mathbb{N}}$ *be generated by Algorithm* 3.1. *Then* $\{x_{n}\}_{n\in\mathbb{N}}$ *converges to* *x̄*, *where* $\Omega_{\mathrm{SDCP}}=\{\bar{x}\}$.

### Proof

Take any $w\in\Omega_{\mathrm{SDCP}}$ and $n\in\mathbb {N}$, and let *w* and *n* be fixed. First, we know that

3.2 $$ 0\in\partial g_{1}(x_{n+1})+ \frac{1}{\beta_{n}}(x_{n+1}-\widehat {x}_{n})-\nabla h_{1}(\widehat{x}_{n}). $$

By (3.2) and Lemma 2.4 we have

3.3 $$ x_{n+1}=(I+\beta_{n}\partial g_{1})^{-1}\bigl(\widehat{x}_{n}+ \beta_{n}\nabla h_{1}(\widehat{x}_{n})\bigr). $$

By (3.2) again, there exists $\tau_{n}\in \partial g_{1}(x_{n+1})$ such that

3.4 $$ \nabla h_{1}(\widehat{x}_{n})= \tau_{n}+\frac{1}{\beta_{n}}(x_{n+1}-\widehat{x}_{n}). $$

Since $w\in\Omega_{\mathrm{SDCP}}$, we have that $\nabla h_{1}(w)\in \partial g_{1}(w)$. By Lemma 2.2, $\partial g_{1}$ is *ρ*-strongly monotone, and this implies that

3.5 $$ 0\leq\bigl\langle x_{n+1}-w,\tau_{n}-\nabla h_{1}(w)\bigr\rangle -\rho \Vert x_{n+1}-w \Vert ^{2}. $$

By (3.4) and (3.5) we have

3.6 $$ \begin{aligned}[b] 0\leq{}& 2\beta_{n}\bigl\langle x_{n+1}-w,\nabla h_{1}(\widehat{x}_{n})- \nabla h_{1}(w)\bigr\rangle -2\beta_{n}\rho \Vert x_{n+1}-w \Vert ^{2} \\ &{} +2\langle x_{n+1}-w,\widehat{x}_{n}-x_{n+1} \rangle \\ \leq{}& 2\beta_{n}L \Vert x_{n+1}-w \Vert \cdot \Vert \widehat{x}_{n}-w \Vert -2\beta_{n}\rho \Vert x_{n+1}-w \Vert ^{2} \\ &{} + \Vert \widehat{x}_{n}-w \Vert ^{2}- \Vert x_{n+1}-\widehat {x}_{n} \Vert ^{2}- \Vert x_{n+1}-w \Vert ^{2} \\ \leq{}& \beta_{n}L\bigl( \Vert x_{n+1}-w \Vert ^{2}+ \Vert \widehat{x}_{n}-w \Vert ^{2}\bigr) -2\beta_{n}\rho \Vert x_{n+1}-w \Vert ^{2} \\ &{} + \Vert \widehat{x}_{n}-w \Vert ^{2}- \Vert x_{n+1}-\widehat{x}_{n} \Vert ^{2}- \Vert x_{n+1}-w \Vert ^{2}. \end{aligned} $$

Hence, by (3.6),

3.7 $$ \Vert x_{n+1}-w \Vert ^{2}\leq \frac{1+\beta_{n}L}{1+2\beta_{n}\rho-\beta _{n}L} \Vert \widehat{x}_{n}-w \Vert ^{2} - \frac{1}{1+2\beta_{n}\rho-\beta _{n}L} \Vert x_{n+1}-\widehat{x}_{n} \Vert ^{2}. $$

Similarly to (3.2), we have

3.8 $$ 0\in\partial g_{2}(\widehat{y}_{n})+ \frac{1}{\beta_{n}}(\widehat {y}_{n}-Aw_{n})-\nabla h_{2}(Aw_{n}) $$

and

3.9 $$ 0\in\partial g_{1}(w_{n})+ \frac{1}{\beta_{n}}(w_{n}-z_{n})-\nabla h_{1}(z_{n}). $$

Similarly to (3.3), we have

3.10 $$ y_{n}=(I+\beta_{n}\partial g_{2})^{-1}\bigl(Ax_{n}+\beta_{n}\nabla h_{2}(Ax_{n})\bigr) $$

and

3.11 $$ \widehat{y}_{n}=(I+\beta_{n}\partial g_{2})^{-1}\bigl(Aw_{n}+\beta_{n}\nabla h_{2}(Aw_{n})\bigr). $$

Similarly to (3.7), we have

3.12 $$\begin{aligned} & \Vert w_{n}-w \Vert ^{2}\leq \frac{1+\beta_{n}L}{1+2\beta_{n}\rho-\beta _{n}L} \Vert z_{n}-w \Vert ^{2} - \frac{1}{1+2\beta_{n}\rho-\beta_{n}L} \Vert w_{n}-z_{n} \Vert ^{2}, \end{aligned}$$

3.13 $$\begin{aligned} & \Vert \widehat{y}_{n}-Aw \Vert ^{2}\leq \frac{\beta_{n}L+1}{1+2\beta_{n}\rho-\beta _{n}L} \Vert Aw_{n}-Aw \Vert ^{2}- \frac{ \Vert \widehat{y}_{n}-Aw_{n} \Vert ^{2}}{1+2\beta_{n}\rho -\beta_{n}L}, \end{aligned}$$

and

3.14 $$ \Vert y_{n}-Aw \Vert ^{2}\leq \frac{\beta_{n}L+1}{1+2\beta_{n}\rho-\beta _{n}L} \Vert Ax_{n}-Aw \Vert ^{2}- \frac{ \Vert y_{n}-Ax_{n} \Vert ^{2}}{1+2\beta_{n}\rho-\beta_{n}L}. $$

Next, we set

3.15 $$ \varepsilon_{n}:=r_{n}\bigl[A^{*}(Aw_{n}- \widehat{y}_{n})-A^{*}(Ax_{n}-y_{n})\bigr]. $$

By (3.10) and (3.11) we have

3.16 $$ \begin{aligned}[b] \Vert \varepsilon_{n} \Vert &\leq r_{n} \Vert A \Vert \bigl( \Vert Aw_{n}-Ax_{n} \Vert + \Vert \widehat {y}_{n}-y_{n} \Vert \bigr) \\ &\leq r_{n} \Vert A \Vert \bigl( \Vert Ax_{n}-Aw_{n} \Vert + \Vert A_{n}-Aw_{n} \Vert +\beta_{n}L \Vert Ax_{n}-Aw_{n} \Vert \bigr) \\ &\leq r_{n} \Vert A \Vert ^{2}(2+\beta_{n}L) \Vert x_{n}-w_{n} \Vert \\ &\leq \sqrt{\delta} \Vert x_{n}-w_{n} \Vert . \end{aligned}$$

By (3.15) we have

3.17 $$ \begin{aligned}[b] \langle x_{n}-w_{n}, D_{n}\rangle={} &\langle x_{n}-w_{n},x_{n}-w_{n}+ \varepsilon_{n}\rangle \\ ={} & \Vert x_{n}-w_{n} \Vert ^{2}+\langle x_{n}-w_{n},\varepsilon_{n}\rangle \\ \geq{}& \Vert x_{n}-w_{n} \Vert ^{2}- \bigl\vert \langle x_{n}-w_{n},\varepsilon_{n}\rangle \bigr\vert \\ \geq{}& (1-\delta) \Vert x_{n}-w_{n} \Vert ^{2} \end{aligned} $$

and

3.18 $$ \begin{aligned}[b] \langle x_{n}-w_{n}, D_{n}\rangle& = \langle x_{n}-w_{n},x_{n}-w_{n}+ \varepsilon _{n}\rangle \\ & = \Vert x_{n}-w_{n} \Vert ^{2}+\langle x_{n}-w_{n},\varepsilon_{n}\rangle \\ & = \frac{1}{2} \Vert x_{n}-w_{n} \Vert ^{2}+\langle x_{n}-w_{n},\varepsilon_{n} \rangle+ \frac{1}{2} \Vert x_{n}-w_{n} \Vert ^{2} \\ & \geq \frac{1}{2} \Vert x_{n}-w_{n} \Vert ^{2}+\langle x_{n}-w_{n},\varepsilon _{n} \rangle+ \frac{1}{2} \Vert \varepsilon_{n} \Vert ^{2} \\ & = \frac{1}{2} \Vert x_{n}-w_{n}+ \varepsilon_{n} \Vert ^{2} \\ & = \frac{1}{2} \Vert D_{n} \Vert ^{2}. \end{aligned} $$

By (3.18) we know that $\alpha_{n}\geq \frac{1}{2}$ for each $n\in\mathbb{N}$. Besides, we have

3.19 $$\begin{aligned} \Vert x_{n}-w_{n}+\varepsilon_{n} \Vert ^{2} = & \Vert x_{n}-w_{n} \Vert ^{2}+ \Vert \varepsilon _{n} \Vert ^{2}+2 \langle x_{n}-w_{n},\varepsilon_{n}\rangle \\ \geq& \Vert x_{n}-w_{n} \Vert ^{2}+ \Vert \varepsilon_{n} \Vert ^{2}-2 \bigl\vert \langle x_{n}-w_{n},\varepsilon_{n}\rangle \bigr\vert \\ \geq& \Vert x_{n}-w_{n} \Vert ^{2}+ \Vert \varepsilon_{n} \Vert ^{2}-2 \Vert x_{n}-w_{n} \Vert \cdot \Vert \varepsilon_{n} \Vert \\ \geq& \Vert x_{n}-w_{n} \Vert ^{2}+ \Vert \varepsilon_{n} \Vert ^{2}-2\delta \Vert x_{n}-w_{n} \Vert ^{2} \\ \geq& (1-2\delta) \Vert x_{n}-w_{n} \Vert ^{2}>0. \end{aligned}$$

By (3.19) we have

3.20 $$ \alpha_{n}^{2}\leq \biggl(\frac{ \Vert x_{n}-w_{n} \Vert \cdot \Vert x_{n}-w_{n}+\varepsilon _{n} \Vert }{ \Vert x_{n}-w_{n}+\varepsilon_{n} \Vert ^{2}} \biggr)^{2} \leq \frac{ \Vert x_{n}-w_{n} \Vert ^{2}}{(1-2\delta) \Vert x_{n}-w_{n} \Vert ^{2}}=\frac {1}{1-2\delta}. $$

Next, we have

3.21 $$\begin{aligned} \Vert \widehat{x}_{n}-w \Vert ^{2} =& \Vert x_{n}-\alpha_{n}D_{n}-w \Vert ^{2} \\ =& \Vert x_{n}-w \Vert ^{2}+\alpha_{n}^{2} \Vert D_{n} \Vert ^{2}-2\alpha_{n}\langle x_{n}-w,D_{n}\rangle \\ =& \Vert x_{n}-w \Vert ^{2}+\alpha_{n}^{2} \Vert D_{n} \Vert ^{2}-2\alpha_{n}\langle x_{n}-w_{n},D_{n}\rangle \\ &{}-2\alpha_{n}\langle w_{n}-w,D_{n}\rangle \\ =& \Vert x_{n}-w \Vert ^{2}-\alpha_{n}^{2} \Vert D_{n} \Vert ^{2}-2\alpha_{n}\langle w_{n}-w,D_{n}\rangle \\ =& \Vert x_{n}-w \Vert ^{2}-\alpha_{n}^{2} \Vert D_{n} \Vert ^{2}-2\alpha_{n}\langle w_{n}-w,z_{n}-\widehat{z}_{n}\rangle \\ =& \Vert x_{n}-w \Vert ^{2}-\alpha_{n}^{2} \Vert D_{n} \Vert ^{2}-\alpha_{n} \Vert w_{n}-\widehat {z}_{n} \Vert ^{2}- \alpha_{n} \Vert z_{n}-w \Vert ^{2} \\ &{}+\alpha_{n} \Vert w_{n}-z_{n} \Vert ^{2}+\alpha_{n} \Vert \widehat{z}_{n}-w \Vert ^{2}. \end{aligned}$$

On the other hand, we have

3.22 $$\begin{aligned} 2 \Vert \widehat{z}_{n}-w \Vert ^{2} =& 2 \bigl\langle \widehat{z}_{n}-w,w_{n}-r_{n} A^{*}(Aw_{n}-\widehat{y}_{n})-w\bigr\rangle \\ =& 2\langle\widehat{z}_{n}-w,w_{n}-w\rangle-2r_{n} \bigl\langle \widehat {z}_{n}-w,A^{*}(Aw_{n}- \widehat{y}_{n})\bigr\rangle \\ =& 2\langle\widehat{z}_{n}-w,w_{n}-w\rangle-2r_{n} \langle A\widehat {z}_{n}-Aw,Aw_{n}-\widehat{y}_{n} \rangle \\ =& \Vert \widehat{z}_{n}-w \Vert ^{2}+ \Vert w_{n}-w \Vert ^{2}- \Vert \widehat {z}_{n}-w_{n} \Vert ^{2}-r_{n} \Vert A\widehat{z}_{n}- \widehat{y}_{n} \Vert ^{2} \\ &{}-r_{n} \Vert Aw_{n}-Aw \Vert ^{2}+r_{n} \Vert A\widehat{z}_{n}-Aw_{n} \Vert ^{2}+r_{n} \Vert \widehat{y}_{n}-Aw \Vert ^{2}, \end{aligned}$$

which implies that

3.23 $$\begin{aligned} \Vert \widehat{z}_{n}-w \Vert ^{2} =& \Vert w_{n}-w \Vert ^{2}- \Vert \widehat {z}_{n}-w_{n} \Vert ^{2}-r_{n} \Vert A\widehat{z}_{n}-\widehat{y}_{n} \Vert ^{2} \\ &{}-r_{n} \Vert Aw_{n}-Aw \Vert ^{2}+r_{n} \Vert A\widehat{z}_{n}-Aw_{n} \Vert ^{2}+r_{n} \Vert \widehat{y}_{n}-Aw \Vert ^{2}. \end{aligned}$$

By (3.12), (3.13), (3.21), and (3.23) we have

3.24 $$\begin{aligned} & \Vert \widehat{x}_{n}-w \Vert ^{2} \\ &\quad = \Vert x_{n}-w \Vert ^{2}-\alpha_{n}^{2} \Vert D_{n} \Vert ^{2}-2\alpha_{n} \Vert w_{n}-\widehat {z}_{n} \Vert ^{2}- \alpha_{n} \Vert z_{n}-w \Vert ^{2} \\ &\qquad {}+\alpha_{n} \Vert w_{n}-z_{n} \Vert ^{2}+\alpha_{n} \Vert w_{n}-w \Vert ^{2}-\alpha_{n}r_{n} \Vert A\widehat {z}_{n}-\widehat{y}_{n} \Vert ^{2} \\ &\qquad {}-\alpha_{n}r_{n} \Vert Aw_{n}-Aw \Vert ^{2}+\alpha_{n}r_{n} \Vert A\widehat {z}_{n}-Aw_{n} \Vert ^{2}+\alpha_{n}r_{n} \Vert \widehat{y}_{n}-Aw \Vert ^{2} \\ &\quad \leq \Vert x_{n}-w \Vert ^{2}- \alpha_{n}^{2} \Vert D_{n} \Vert ^{2}- \alpha _{n}\bigl(2-r_{n} \Vert A \Vert ^{2} \bigr) \Vert \widehat{z}_{n}-w_{n} \Vert ^{2}- \alpha_{n} \Vert z_{n}-w \Vert ^{2} \\ &\qquad {}+\alpha_{n} \Vert w_{n}-z_{n} \Vert ^{2}+\alpha_{n} \Vert w_{n}-w \Vert ^{2}-\alpha_{n}r_{n} \Vert A\widehat {z}_{n}-\widehat{y}_{n} \Vert ^{2} \\ &\qquad {}-\alpha_{n}r_{n} \Vert Aw_{n}-Aw \Vert ^{2}+\alpha_{n}r_{n} \Vert \widehat{y}_{n}-Aw \Vert ^{2} \\ &\quad \leq \Vert x_{n}-w \Vert ^{2}- \alpha_{n}^{2} \Vert D_{n} \Vert ^{2}- \alpha _{n}\bigl(2-r_{n} \Vert A \Vert ^{2} \bigr) \Vert \widehat{z}_{n}-w_{n} \Vert ^{2}- \alpha_{n} \Vert z_{n}-w \Vert ^{2} \\ &\qquad {}+\alpha_{n} \Vert w_{n}-z_{n} \Vert ^{2}+\alpha_{n} \Vert z_{n}-w \Vert ^{2}-\frac{\alpha _{n}}{1+2\beta_{n}\rho-\beta_{n}L} \Vert w_{n}-z_{n} \Vert ^{2} \\ &\qquad {}-\alpha_{n}r_{n} \Vert A\widehat{z}_{n}- \widehat{y}_{n} \Vert ^{2}-\alpha _{n}r_{n} \Vert Aw_{n}-Aw \Vert ^{2}+\alpha_{n}r_{n} \Vert Aw_{n}-Aw \Vert ^{2} \\ &\quad \leq \Vert x_{n}-w \Vert ^{2}- \alpha_{n}^{2} \Vert D_{n} \Vert ^{2}- \alpha _{n}\bigl(2-r_{n} \Vert A \Vert ^{2} \bigr) \Vert \widehat{z}_{n}-w_{n} \Vert ^{2} \\ &\qquad {}+\alpha_{n} \Vert w_{n}-z_{n} \Vert ^{2}-\frac{\alpha_{n}}{1+2\beta_{n}\rho-\beta _{n}L} \Vert w_{n}-z_{n} \Vert ^{2} \\ &\qquad {}-\alpha_{n}r_{n} \Vert A\widehat{z}_{n}- \widehat{y}_{n} \Vert ^{2}. \end{aligned}$$

We also have

3.25 $$ -2\alpha_{n}^{2} \Vert D_{n} \Vert ^{2}=\alpha_{n} \Vert w_{n}- \widehat{z}_{n} \Vert ^{2}+\alpha _{n} \Vert x_{n}-z_{n} \Vert ^{2}-\alpha_{n} \Vert w_{n}-z_{n} \Vert ^{2}- \alpha_{n} \Vert x_{n}-\widehat{z}_{n} \Vert ^{2}. $$

By (3.24) and (3.25) we have

3.26 $$\begin{aligned} & \Vert \widehat{x}_{n}-w \Vert ^{2} \\ &\quad \leq \Vert x_{n}-w \Vert ^{2}- \alpha_{n}\biggl(\frac{3}{2}-r_{n} \Vert A \Vert ^{2}\biggr) \Vert \widehat {z}_{n}-w_{n} \Vert ^{2}-\alpha_{n}r_{n} \Vert A\widehat{z}_{n}- \widehat{y}_{n} \Vert ^{2} \\ &\qquad {}-\frac{\alpha_{n}}{1+2\beta_{n}\rho-\beta_{n}L} \Vert w_{n}-z_{n} \Vert ^{2}-\frac {1}{2}\cdot\alpha_{n} \Vert x_{n}-\widehat{z}_{n} \Vert ^{2}+ \frac{1}{2}\cdot\alpha _{n} \Vert x_{n}-z_{n} \Vert ^{2} \\ &\qquad {}+\frac{1}{2}\cdot\alpha_{n} \Vert w_{n}-z_{n} \Vert ^{2}. \end{aligned}$$

By (3.14) we have

3.27 $$\begin{aligned} \Vert x_{n}-z_{n} \Vert =& \bigl\Vert r_{n}A^{*}(Ax_{n}-y_{n}) \bigr\Vert \\ \leq& r_{n} \Vert A \Vert \bigl( \Vert Ax_{n}-Aw \Vert + \Vert y_{n}-Aw \Vert \bigr) \\ \leq& 2r_{n} \Vert A \Vert \cdot \Vert Ax_{n}-Aw \Vert \\ \leq& 2r_{n} \Vert A \Vert ^{2} \Vert x_{n}-w \Vert . \end{aligned}$$

By (3.7), (3.26), and (3.27) we have

3.28 $$\begin{aligned} \Vert x_{n+1}-w \Vert ^{2} \leq& \frac{1+\beta_{n}L}{1+2\beta_{n}\rho-\beta_{n}L} \Vert \widehat {x}_{n}-w \Vert ^{2} - \frac{1}{1+2\beta_{n}\rho-\beta_{n}L} \Vert x_{n+1}-\widehat {x}_{n} \Vert ^{2} \\ \leq& \frac{1+\beta_{n}L}{1+2\beta_{n}\rho-\beta_{n}L} \biggl( \Vert x_{n}-w \Vert ^{2}-\alpha_{n}\biggl(\frac{3}{2}-r_{n} \Vert A \Vert ^{2}\biggr) \Vert \widehat{z}_{n}-w_{n} \Vert ^{2} \\ &{}-\alpha_{n}r_{n} \Vert A\widehat{z}_{n}- \widehat{y}_{n} \Vert ^{2}-\alpha_{n}\cdot \biggl( \frac{1}{1+2\beta_{n}\rho-\beta_{n}L}-\frac{1}{2}\biggr) \Vert w_{n}-z_{n} \Vert ^{2} \\ &{}-\frac{1}{2}\cdot\alpha_{n} \Vert x_{n}- \widehat{z}_{n} \Vert ^{2}+\frac {1}{2}\cdot \alpha_{n} \Vert x_{n}-z_{n} \Vert ^{2} \biggr) \\ &{}-\frac{1}{1+2\beta_{n}\rho-\beta_{n}L} \Vert x_{n+1}-\widehat{x}_{n} \Vert ^{2} \\ \leq& \frac{1+\beta_{n}L}{1+2\beta_{n}\rho-\beta_{n}L}\bigl(1+2\alpha _{n}r_{n}^{2} \Vert A \Vert ^{4}\bigr) \Vert x_{n}-w \Vert ^{2} \\ \leq& \Vert x_{n}-w \Vert ^{2}. \end{aligned}$$

By (3.28), $\lim_{n\rightarrow \infty }\|x_{n}-w\|$ exists, $\{x_{n}\}_{n\in\mathbb{N}}$ is a bounded sequence, and

3.29 $$ \textstyle\begin{cases} \lim_{n\rightarrow \infty}\frac{1+\beta_{n}L}{1+2\beta_{n}\rho-\beta _{n}L}\cdot\alpha_{n}(\frac{3}{2}-r_{n} \Vert A \Vert ^{2}) \Vert \widehat{z}_{n}-w_{n} \Vert ^{2}=0, \\ \lim_{n\rightarrow \infty}\frac{1+\beta_{n}L}{1+2\beta_{n}\rho-\beta _{n}L}\cdot\alpha_{n}r_{n} \Vert A\widehat{z}_{n}-\widehat{y}_{n} \Vert ^{2}=0, \\ \lim_{n\rightarrow \infty}\frac{1+\beta_{n}L}{1+2\beta_{n}\rho-\beta _{n}L}\cdot\frac{\alpha_{n}}{1+2\beta_{n}\rho-\beta_{n}L} \Vert w_{n}-z_{n} \Vert ^{2}=0, \\ \lim_{n\rightarrow \infty}\frac{1+\beta_{n}L}{1+2\beta_{n}\rho-\beta _{n}L}\cdot\frac{1}{2}\cdot\alpha_{n} \Vert x_{n}-\widehat{z}_{n} \Vert ^{2}=0. \end{cases} $$

By the assumptions we have

3.30 $$ \lim_{n\rightarrow \infty} \Vert \widehat{z}_{n}-w_{n} \Vert =\lim_{n\rightarrow \infty} \Vert A\widehat{z}_{n}- \widehat{y}_{n} \Vert =\lim_{n\rightarrow \infty} \Vert w_{n}-z_{n} \Vert =\lim_{n\rightarrow \infty } \Vert x_{n}-\widehat{z}_{n} \Vert =0. $$

Since $\{x_{n}\}_{n\in\mathbb{N}}$ is bounded, there exists a subsequence $\{x_{n_{k}}\}_{k\in\mathbb{N}}$ of $\{x_{n}\}_{n\in\mathbb {N}}$ such that $x_{n_{k}}\rightarrow \bar{x}\in H_{1}$. Thus, $w_{n_{k}}\rightarrow \bar{x}$, $z_{n_{k}}\rightarrow \bar{x}$, $Aw_{n_{k}}\rightarrow A\bar{x}$, and $\widehat{y}_{n_{k}}\rightarrow A\bar{x}$. By (3.8), (3.9), and Lemma 2.3 we get that $\bar{x}\in\Omega _{\text{SDCP}}$. By Proposition 3.1, $\Omega_{\text{SDCP}}=\{\bar{x}\}$. Further, $\lim_{n\rightarrow \infty }\|x_{n}-\bar{x}\|=\lim_{k\rightarrow \infty}\|x_{n_{k}}-\bar{x}\|=0$. Therefore the proof is completed. □

## Main results in infinite-dimensional real Hilbert space

Let $H_{1}$ and $H_{2}$ be infinite-dimensional real Hilbert spaces. Let *δ*, *ρ*, *L*, *A*, $A^{*}$, $g_{1}$, $h_{1}$, $g_{2}$, $h_{2}$, $f_{1}$, $f_{2}$, $\{r_{n}\}_{n\in\mathbb{N}}$, and $\{\beta_{n}\}_{n\in\mathbb {N}}$ be the same as in Sect. 3.

### Definition 4.1

Let *C* be a nonempty closed convex subset of a real Hilbert space *H*, and let $T:C\rightarrow H$. Let $\operatorname{Fix}(T):=\{x\in C: Tx=x\}$. Then:

(i) *T* is a nonexpansive mapping if $\|Tx-Ty\|\leq\|x-y\|$ for all $x,y\in C$;
(ii) *T* is a firmly nonexpansive mapping if $\|Tx-Ty\|^{2}\leq \langle x-y,Tx-Ty\rangle$ for all $x,y\in C$, that is, $\|Tx-Ty\|^{2}\leq\|x-y\|^{2}-\|(I-T)x-(I-T)y\|^{2}$ for all $x,y\in C$.

### Lemma 4.1

([21])

*Let* *C* *be a nonempty closed convex subset of a real Hilbert space H*. *Let* $T:C\rightarrow H$ *be a nonexpansive mapping*, *and let* $\{x_{n}\} _{n\in\mathbb{N}}$ *be a sequence in* *C*. *If* $x_{n}\rightharpoonup w$ *and* $\lim_{n\rightarrow \infty}\|x_{n}-Tx_{n}\|=0$, *then* $Tw=w$.

### Definition 4.2

Let $\beta>0$, let *H* be a real Hilbert space, and let $g:H\rightarrow \mathbb{R}$ be a proper lower-semicontinuous and convex function. Then the proximal operator of *g* of order *β* is defined by

$$\operatorname{prox}_{\beta,g}(x):=\mathop{\operatorname{argmin}}_{v\in H}\biggl\{ g(v)+ \frac{1}{2\beta } \Vert v-x \Vert ^{2}\biggr\} $$

for each $x\in H$. In fact, we know that $\operatorname{prox}_{\beta,g}(x)=(I+\beta\partial g)^{-1}(x)=J_{\beta}^{\partial g}(x)$ and $T(x):=\operatorname{prox}_{\beta,g}(x)$ is a firmly nonexpansive mapping.

### Lemma 4.2

([22, Lemma 2.3])

*Let* *H* *be a real Hilbert space*, *and let* $g:H\rightarrow \mathbb{R}$ *be a proper lower*-*semicontinuous and convex function*. *For* $\beta_{2}\geq \beta_{1}>0$, *we have*

$$\operatorname{prox}_{\beta_{2},g}(x)=\operatorname{prox}_{\beta_{1},g}\bigg(\frac{\beta_{1}}{\beta_{2}}x+(1-\frac{\beta_{1}}{\beta_{2}})\operatorname{prox}_{\beta_{2},g}(x)\bigg). $$

The following result plays an important role when we study our convergence theorem in an infinite-dimensional real Hilbert space.

### Lemma 4.3

*Let* *H* *be a real Hilbert space*, *let* $g, h:H\rightarrow \mathbb {R}$ *be proper lower*-*semicontinuous and convex functions*, *and suppose that* *h* *is Fréchet differentiable*. *Then for all* $x\in H$ *and* $0<\beta_{1}\leq\beta_{2}$, *we have*

$$\bigl\| x-\operatorname{prox}_{\beta_{1},g}(x+\beta_{1}\nabla h(x))\bigr\| \leq 2 \bigl\| x-\operatorname{prox}_{\beta_{2},g}(x+\beta_{2}\nabla h(x))\bigr\| . $$

### Proof

By Lemma 4.2 we have

$$ \operatorname{prox}_{\beta_{2},g}\bigl(x+\beta_{2}\nabla h(x)\bigr)=\operatorname{prox}_{\beta_{1},g} \biggl(\frac {\beta_{1}}{\beta_{2}}\bigl(x+\beta_{2} \nabla h(x)\bigr)+\biggl(1-\frac{\beta_{1}}{\beta _{2}}\biggr)\operatorname{prox}_{\beta_{2},g} \bigl(x+\beta_{2}\nabla h(x)\bigr) \biggr). $$

Thus,

$$\begin{aligned} & \bigl\Vert \operatorname{prox}_{\beta_{1},g}\bigl(x+\beta_{1}\nabla h(x)\bigr)-\operatorname{prox}_{\beta_{2},g}\bigl(x+\beta _{2}\nabla h(x) \bigr) \bigr\Vert \\ &\quad \leq \biggl\| x+\beta_{1}\nabla h(x)- \biggl(\frac{\beta_{1}}{\beta _{2}} \bigl(x+\beta_{2}\nabla h(x)\bigr)+\biggl(1-\frac{\beta_{1}}{\beta_{2}}\biggr) \operatorname{prox}_{\beta _{2},g}\bigl(x+\beta_{2}\nabla h(x)\bigr) \biggr)\biggr\| \\ &\quad = \biggl(1-\frac{\beta_{1}}{\beta_{2}}\biggr) \bigl\Vert x-\operatorname{prox}_{\beta_{2},g} \bigl(x+\beta_{2}\nabla h(x)\bigr) \bigr\Vert \\ &\quad \leq \bigl\Vert x-\operatorname{prox}_{\beta_{2},g}\bigl(x+ \beta_{2}\nabla h(x)\bigr) \bigr\Vert , \end{aligned}$$

and then

$$\begin{aligned} & \bigl\Vert x-\operatorname{prox}_{\beta_{1},g}\bigl(x+\beta_{1}\nabla h(x)\bigr) \bigr\Vert \\ &\quad \leq \bigl\Vert x-\operatorname{prox}_{\beta_{2},g}\bigl(x+ \beta_{2}\nabla h(x)\bigr) \bigr\Vert + \bigl\Vert \operatorname{prox}_{\beta _{2},g}\bigl(x+\beta_{2}\nabla h(x)\bigr)- \operatorname{prox}_{\beta_{1},g}\bigl(x+\beta_{1}\nabla h(x)\bigr) \bigr\Vert \\ &\quad \leq 2 \bigl\Vert x-\operatorname{prox}_{\beta_{2},g}\bigl(x+ \beta_{2}\nabla h(x)\bigr) \bigr\Vert . \end{aligned}$$

Therefore the proof is completed. □

### Lemma 4.4

*Let* $\beta>0$, *let* *H* *be a real Hilbert space*, *and let* $g:H\rightarrow \mathbb{R}$ *be a proper lower semicontinuous and* *ρ*-*strongly convex function*. *Then* $T(x):=\operatorname{prox}_{\beta,g}(x)$ *is a contraction mapping*. *In fact*, $\|Tx-Ty\|\leq\frac{1}{1+\beta\rho}\|x-y\|$.

### Lemma 4.5

*Let* $\beta>0$, *let* *H* *be a real Hilbert space*, *and let* $g, h:H\rightarrow \mathbb{R}$ *be proper lower semicontinuous and convex functions*. *Further*, *we assume that* *h* *is Fréchet differentiable*, ∇*h* *is* *L*-*Lipschitz continuous*, *and* *g* *is* *ρ*-*strongly convex*. *Let* $T:H\rightarrow H$ *be defined by* $Tx:=\operatorname{prox}_{\beta ,g}(x+\beta\nabla h(x))$ *for each* $x\in H$. *Then the following are satisfied*.

(i) *If* $\rho>L>0$, *then* *T* *is a contraction mapping*.
(ii) *If* $\rho=L>0$, *then* *T* *is a nonexpansive mapping*.

### Proof

For $x,y\in H$, we have

$$\begin{aligned} \Vert Tx-Ty \Vert \leq& \frac{1}{1+\beta\rho} \bigl\Vert \bigl(x+\beta\nabla h(x)\bigr)-\bigl(y+\beta\nabla h(y)\bigr) \bigr\Vert \\ \leq& \frac{1}{1+\beta\rho} \bigl( \Vert x-y \Vert +\beta \bigl\Vert \nabla h(x)-\nabla h(y)\bigr) \bigr\Vert ) \\ \leq& \frac{1}{1+\beta\rho} \bigl( \Vert x-y \Vert +\beta L \Vert x-y \Vert \bigr) \\ =& \frac{1+\beta L}{1+\beta\rho} \Vert x-y \Vert . \end{aligned}$$

Thus the proof is completed. □

### Theorem 4.1

*In Theorem* 3.1, *let* $H_{1}$ *and* $H_{2}$ *be an infinite*-*dimensional real Hilbert space and assume that* $\liminf_{n\rightarrow \infty}\beta_{n}>0$. *Then the sequence* $\{x_{n}\}_{n\in\mathbb{N}}$ *generated by Algorithm* 3.1 *converges weakly to the unique solution* *x̄* *of problem* (SDCP).

### Proof

By Proposition 3.1 we know that $\Omega_{\mathrm{SDCP}}=\{\bar{x}\}$. Since $\liminf_{n\rightarrow \infty}\beta_{n}>0$, we may assume that there exists a real number $\beta^{*}$ such that $\beta_{n}>\beta^{*}>0$. By (3.11) we have

4.1 $$ \widehat{y}_{n}=(I+\beta_{n}\partial g_{2})^{-1}\bigl(Aw_{n}+\beta_{n}\nabla h_{2}(Aw_{n})\bigr)=\operatorname{prox}_{\beta_{n},g_{2}} \bigl(Aw_{n}+\beta_{n}\nabla h_{2}(Aw_{n}) \bigr). $$

Similarly, we have

4.2 $$ w_{n}=(I+\beta_{n}\partial g_{1})^{-1}\bigl(z_{n}+\beta_{n}\nabla h_{1}(z_{n})\bigr)=\operatorname{prox}_{\beta_{n},g_{1}} \bigl(z_{n}+\beta_{n}\nabla h_{1}(z_{n}) \bigr). $$

By (3.30) we know that

4.3 $$ \lim_{n\rightarrow \infty} \Vert Aw_{n}- \widehat{y}_{n} \Vert =\lim_{n\rightarrow \infty} \Vert w_{n}-z_{n} \Vert =\lim_{n\rightarrow \infty} \Vert x_{n}-w_{n} \Vert =0. $$

By (4.2) and (4.3) we have

4.4 $$ \lim_{n\rightarrow \infty} \bigl\Vert z_{n}- \operatorname{prox}_{\beta_{n},g_{1}}\bigl(z_{n}+\beta _{n}\nabla h_{1}(z_{n})\bigr) \bigr\Vert =0 $$

and

4.5 $$ \lim_{n\rightarrow \infty} \bigl\Vert Aw_{n}- \operatorname{prox}_{\beta_{n},g_{2}}\bigl(Aw_{n}+\beta _{n}\nabla h_{2}(Aw_{n})\bigr) \bigr\Vert =0. $$

By (4.4), (4.5), and Lemma 4.3 we have

4.6 $$ \lim_{n\rightarrow \infty} \bigl\Vert z_{n}- \operatorname{prox}_{\beta^{*},g_{1}}\bigl(z_{n}+\beta ^{*}\nabla h_{1}(z_{n})\bigr) \bigr\Vert =0 $$

and

4.7 $$ \lim_{n\rightarrow \infty} \bigl\Vert Aw_{n}- \operatorname{prox}_{\beta^{*},g_{2}}\bigl(Aw_{n}+\beta ^{*}\nabla h_{2}(Aw_{n})\bigr) \bigr\Vert =0. $$

Besides, we have to show that $\{x_{n}\}_{n\in\mathbb{N}}$ is a bounded sequence. Since $H_{1}$ is infinite dimensional, there exist $\bar{x}\in H_{1}$ and a subsequence $\{x_{n_{k}}\}_{k\in\mathbb{N}}$ of $\{x_{n}\} _{n\in\mathbb{N}}$ such that $x_{n_{k}}\rightharpoonup x^{*}\in H_{1}$. By (4.3) we know that $z_{n_{k}}\rightharpoonup x^{*}$ and $w_{n_{k}}\rightharpoonup x^{*}$. Hence, by (4.6), Lemma 4.1, and Lemma 4.5 we have that $x^{*}=\operatorname{prox}_{\beta^{*},g_{1}}(x^{*}+\beta^{*}\nabla h_{1}(x^{*}))$, which implies that $\nabla h_{1}(x^{*})\in\partial g_{1}(x^{*})$. Since *A* is linear, we have $Aw_{n_{k}}\rightharpoonup Ax^{*}$. Hence, by (4.7), Lemma 4.1 and Lemma 4.5, we have $Ax^{*}=\operatorname{prox}_{\beta^{*},g_{2}}(Ax^{*}+\beta^{*}\nabla h_{2}(Ax^{*}))$, which implies that $\nabla h_{2}(Ax^{*})\in\partial g_{2}(Ax^{*})$. So, $x^{*}\in\Omega_{\mathrm{SDCP}}$, and thus $\lim_{n\rightarrow \infty}\|x_{n}-x^{*}\|$ exists. So, by Opial’s condition, we get $x_{n}\rightharpoonup x^{*}$. Therefore the proof is completed. □

### Remark 4.1

To the best of our knowledge, the convergence theorems for the DC program and split DC program are proposed in finite-dimensional Hilbert spaces. Here, Theorem 4.1 is a convergence theorem for the split DC program in infinite-dimensional real Hilbert spaces.

Following the same argument as in the proof of Theorem 4.1, we get the following convergence theorem in infinite-dimensional real Hilbert spaces.

### Theorem 4.2

*Let* $H_{1}$ *and* $H_{2}$ *be infinite*-*dimensional real Hilbert spaces*. *Let* *A*, $A^{*}$, $g_{1}$, $h_{1}$, $g_{2}$, $h_{2}$, $f_{1}$, *and* $f_{2}$ *be the same as in Sect*. 3. *Let* $\rho\geq L>0$. *Let* $\{\beta_{n}\}_{n\in\mathbb{N}}$ *be a sequence in* $[a,b]\subseteq(0,\infty)$. *Let* $\{r_{n}\}_{n\in\mathbb {N}}$ *be a sequence in* $(0,\frac{1}{\|A\|^{2}})$ *such that* $0<\liminf_{n\rightarrow \infty}r_{n}\leq\limsup_{n\rightarrow \infty}r_{n}<\frac{1}{\|A\|^{2}}$. *Then the sequence* $\{x_{n}\}_{n\in \mathbb{N}}$ *generated by Algorithm* 1.3 *converges weakly to some* $\bar{x}\in\Omega_{\mathrm{SDCP}}$.

## Application to DC program

Let *ρ*, *L*, *δ*, $\{\beta_{n}\}_{n\in\mathbb{N}}$ be the same as in Sect. 3. Let *H* be an infinite-dimensional Hilbert space, and let $g,h:H\rightarrow \mathbb{R}$ be proper lower semicontinuous and convex functions. Besides, we also assume that *h* is Fréchet differentiable, ∇*h* is *L*-Lipschitz continuous, and *g* is *ρ*-strongly convex. Let $f(x)=g(x)-h(x)$ for all $x\in H$ and assume that *f* is bounded from below.

Let $\{r_{n}\}_{n\in\mathbb{N}}$ be a sequence in $\mathbb{R}$ with $\liminf_{n\rightarrow \infty}r_{n}>0$ and

$$0< r_{n}< \min \biggl\{ \frac{\sqrt[4]{1-2\delta}\cdot\sqrt{\beta _{n}(\rho-L)}}{\sqrt{2+2\beta_{n} L}},\frac{\sqrt{\delta}}{(2+\beta _{n}L)} \biggr\} . $$

Let $\Omega_{\text{DCP}}$ be defined by

$$\Omega_{\text{DCP}}:=\bigl\{ x\in H: \nabla h(x)\in\partial g(x)\bigr\} , $$

and assume that $\Omega_{\text{DCP}}\neq\emptyset$.

The following algorithm and convergence theorem are given by Algorithm 3.1 and Theorem 4.1, respectively.

### Algorithm 5.1

Let $x_{1}\in H$ be arbitrary, and let $\{x_{n}\}_{n\in\mathbb{N}}$ be generated as follows:

$$\textstyle\begin{cases} y_{n}:=\arg\min_{v\in H}\{g(v)+\frac{1}{2\beta_{n}} \Vert v-x_{n} \Vert ^{2}-\langle \nabla h(x_{n}),v-x_{n}\rangle\},\\ z_{n}:=x_{n}-r_{n} (x_{n}-y_{n}),\\ w_{n}:=\arg\min_{u\in H_{1}}\{g(u)+\frac{1}{2\beta _{n}} \Vert u-z_{n} \Vert ^{2}-\langle\nabla h(z_{n}),u-z_{n}\rangle\},\\ \widehat{y}_{n}:=\arg\min_{v\in H}\{g(v)+\frac{1}{2\beta _{n}} \Vert v-w_{n} \Vert ^{2}-\langle\nabla h(w_{n}),v-w_{n}\rangle\},\\ \widehat{z}_{n}:=w_{n}-r_{n} (w_{n}-\widehat{y}_{n}),\\ D_{n}:=z_{n}-\widehat{z}_{n},\\ \alpha_{n}:=\frac{\langle x_{n}-w_{n},D_{n}\rangle}{ \Vert D_{n} \Vert ^{2}},\\ \widehat{x}_{n}:=x_{n}-\alpha_{n}D_{n},\\ x_{n+1}:=\arg\min_{u\in H}\{g(u)+\frac{1}{2\beta_{n}} \Vert u-\widehat {x}_{n} \Vert ^{2}-\langle\nabla h(\widehat{x}_{n}),u-\widehat{x}_{n}\rangle\},\quad n\in\mathbb{N},\\ \text{stop criteria: } x_{n}=w_{n}. \end{cases} $$

### Theorem 5.1

*Assume that* $\liminf_{n\rightarrow \infty}\beta_{n}>0$. *Then the sequence* $\{x_{n}\}_{n\in\mathbb{N}}$ *generated by Algorithm* 5.1 *converges weakly to the unique solution* *x̄* *of problem* (SDCP).

The following algorithm is a particular case of Algorithm 1.3.

### Algorithm 5.2

([13])

Let $x_{1}\in H$ be arbitrary, and let $\{ x_{n}\}_{n\in\mathbb{N}}$ be generated as follows:

$$\textstyle\begin{cases} y_{n}:=\arg\min_{v\in H}\{g(v)+\frac{1}{2\beta_{n}} \Vert v-x_{n} \Vert ^{2}-\langle \nabla h(x_{n}),v-x_{n}\rangle\},\\ z_{n}:=(1-r_{n})x_{n}+r_{n}y_{n},\\ x_{n+1}:=\arg\min_{u\in H}\{g(u)+\frac{1}{2\beta _{n}} \Vert u-z_{n} \Vert ^{2}-\langle\nabla h(z_{n}),u-z_{n}\rangle\},\quad n\in\mathbb{N}. \end{cases} $$

By Theorem 4.2 we get the following result, which it is a generalization of [13, Thm. 4.1].

### Theorem 5.2

*Let* $\rho\geq L>0$. *Let* $\{\beta_{n}\}_{n\in\mathbb{N}}$ *be a sequence in* $[a,b]\subseteq(0,\infty)$. *Let* $\{r_{n}\}_{n\in\mathbb {N}}$ *be a sequence in* $(0,1)$ *such that* $0<\liminf_{n\rightarrow \infty}r_{n}\leq\limsup_{n\rightarrow \infty}r_{n}<1$. *Let* $\{x_{n}\}_{n\in\mathbb{N}}$ *be generated by Algorithm* 5.2. *Then* $\{x_{n}\}_{n\in\mathbb{N}}$ *converges weakly to some* $\bar{x}\in \Omega_{\mathrm{DCP}}$.

Next, we can get the following algorithm and convergence theorem by Algorithm 5.2 and Theorem 5.2, respectively. Further, Theorem 5.3 is a generalization of [13, Thm. 4.2].

### Algorithm 5.3

([13])

Let $x_{1}\in H$ be arbitrary, and let $\{ x_{n}\}_{n\in\mathbb{N}}$ be generated as follows:

$$\textstyle\begin{cases} z_{n}:=\arg\min_{u\in H}\{g(u)+\frac{1}{2\beta _{n}} \Vert u-x_{n} \Vert ^{2}-\langle\nabla h(x_{n}),u-x_{n}\rangle\},\\ y_{n}:=\arg\min_{v\in H}\{g(v)+\frac{1}{2\beta _{n}} \Vert v-z_{n} \Vert ^{2}-\langle\nabla h(z_{n}),v-z_{n}\rangle\},\\ x_{n+1}:=(1-r_{n})z_{n}+r_{n}y_{n},\quad n\in\mathbb{N}. \end{cases} $$

### Theorem 5.3

*Let* $\rho\geq L>0$. *Let* $\{\beta_{n}\}_{n\in\mathbb{N}}$ *be a sequence in* $[a,b]\subseteq(0,\infty)$. *Let* $\{r_{n}\}_{n\in\mathbb {N}}$ *be a sequence in* $(0,1)$ *such that* $0<\liminf_{n\rightarrow \infty}r_{n}\leq\limsup_{n\rightarrow \infty}r_{n}<1$. *Let* $\{x_{n}\}_{n\in\mathbb{N}}$ *be generated by Algorithm* 5.3. *Then* $\{x_{n}\}_{n\in\mathbb{N}}$ *converges weakly to some* $\bar{x}\in \Omega_{\mathrm{DCP}}$.

If $r_{n}=0$ for all $n\in\mathbb{N}$, then we have the following result.

### Theorem 5.4

*Let* $\rho\geq L>0$. *Let* $\{\beta_{n}\}_{n\in\mathbb{N}}$ *be a sequence in* $[a,b]\subseteq(0,\infty)$. *Let* $x_{1}\in H$ *be arbitrary*, *and let* $\{x_{n}\}_{n\in\mathbb{N}}$ *be generated by*

$$x_{n+1}:=\arg\min_{u\in H}\biggl\{ g(u)+ \frac{1}{2\beta _{n}} \Vert u-x_{n} \Vert ^{2}-\bigl\langle \nabla h(x_{n}),u-x_{n}\bigr\rangle \biggr\} ,\quad n\in \mathbb{N}. $$

*Then* $\{x_{n}\}_{n\in\mathbb{N}}$ *converges weakly to some* $\bar{x}\in \Omega_{\mathrm{DCP}}$.

### Proof

Following similar argument as in the proof of Theorem 4.1, we get the statement of Theorem 5.4. □
